# Imaging techniques in diagnostics, differentiation and monitoring of different types of thyroiditis

**DOI:** 10.1186/1756-6614-6-S2-A50

**Published:** 2013-04-05

**Authors:** Marek Ruchała, Ewelina Szczepanek-Parulska

**Affiliations:** 1 Department of Endocrinology, Metabolism and Internal Medicine, Poznan University of Medical Sciences, Poznan, Poland

## 

Thyroid imaging is nowadays an essential part of thyroid disease evaluation. Different types of thyroiditis may share some parallel clinical and biochemical features, what might lead to diagnostic difficulties, while thyroid imaging may aid in establishing proper diagnosis. In the lecture, usefulness of several imaging techniques (conventional ultrasonography, Doppler examination, sonoelastography, scintigraphy and other) in diagnostics, differentiation and monitoring of different types of thyroiditis are presented according to up-to-date guidelines.

The most commonly applied modality in evaluation of thyroiditis is conventional ultrasonography. Chronic autoimmune thyroiditis typically presents with decreased, heterogeneous echogenicity of the whole gland, signs of fibrosis and decreased blood flow in Doppler examination. More often than in other types, in silent thyroiditis the most hypoechogenic area is the anterior part of the thyroid. Subacute thyroiditis presents with an enlargement of the gland, especially in depth. The ill-defined hypoechogenic regions of thyroid parenchyma of different size and shape, change smoothly into those of normal echogenicity. Most commonly, one lobe is involved. Anti-inflammatory treatment results in remission with no residual changes. Initially, acute thyroiditis resembles subacute type. However, gradual resolution can be observed with formation of abscesses with dense content. On ultrasound picture, Riedel’s thyroiditis presents as a hypoechogenic region with ill-defined margins and marked fibrosis. Signs of trachea compression and displacement may be seen. Moreover, features of post-radioiodine and most common drug-induced thyroiditis are presented. These usually present with heterogeneous decreased echogenicity and signs of fibrosis, while may differ according to blood flow picture.

Lately, sonoelastography appeared to be useful in evaluation of various types of thyroiditis. Acute and subacute thyroiditis were found to be associated with decreased elasticity of thyroid tissue, which is restored to normal with treatment. Chronic thyroiditis is associated with a minimally decreased elasticity of thyroid parenchyma, which remains unchanged during therapy. Importantly, coexistence of thyroiditis in certain cases may influence the result of sonoelastographic evaluation of focal lesions. Thyroiditis, depending on the type, may present with focally or diffusely decreased radioisotope uptake in thyroid scintiscan. Experimental use of other modalities, including MR, is also discussed.

